# Challenges of refractive cataract surgery in the era of myopia epidemic: a mini-review

**DOI:** 10.3389/fmed.2023.1128818

**Published:** 2023-09-19

**Authors:** Yu Du, Jiaqi Meng, Wenwen He, Yi Lu, Xiangjia Zhu

**Affiliations:** ^1^Eye Institute and Department of Ophthalmology, Eye & ENT Hospital, Fudan University, Shanghai, China; ^2^NHC Key Laboratory of Myopia, Fudan University, Shanghai, China; ^3^Key Laboratory of Myopia, Chinese Academy of Medical Sciences, Shanghai, China; ^4^Shanghai Key Laboratory of Visual Impairment and Restoration, Shanghai, China; ^5^State Key Laboratory of Medical Neurobiology, Fudan University, Shanghai, China

**Keywords:** myopia epidemic, refractive surgery, cataract, biometry, IOL power calculation, complications

## Abstract

Myopia is the leading cause of visual impairment in the world. With ever-increasing prevalence in these years, it creates an alarming global epidemic. In addition to the difficulty in seeing distant objects, myopia also increases the risk of cataract and advances its onset, greatly affecting the productivity of myopes of working age. Cataract management in myopic eyes, especially highly myopic eyes is originally more complicated than that in normal eyes, whereas the growing population of cataract with myopia, increasing popularity of corneal and lens based refractive surgery, and rising demand for spectacle independence after cataract surgery all further pose unprecedented challenges to ophthalmologists. Previous history of corneal refractive surgery and existence of implantable collamer lens will both affect the accuracy of biometry including measurement of corneal curvature and axial length before cataract surgery, which may result in larger intraocular lens (IOL) power prediction errors and a compromise in the surgical outcome especially in a refractive cataract surgery. A prudent choice of formula for cataract patients with different characteristics is essential in improving this condition. Besides, the characteristics of myopic eyes might affect the long-term stability of IOL, which is important for the maintenance of visual outcomes especially after the implantation of premium IOLs, thus a proper selection of IOL accordingly is crucial. In this mini-review, we provide an overview of the impact of myopia epidemic on treatment for cataract and to discuss new challenges that surgeons may encounter in the foreseeable future when planning refractive cataract surgery for myopic patients.

## Introduction

The myopia epidemic is a major public health concern worldwide, and is particularly notable in East Asia. Over 80% of teenagers in East Asian countries and one-third in Europe and the United States of America (USA) are myopic (1). This prevalence is expected to increase over time due to modern lifestyles and increasing near-work activities, and the COVID-19 pandemic exacerbated the onset and progression of myopia (2–4). Myopia is not just a simple benign condition, and the socioeconomic consequences of myopia extend beyond the cost of its prevention and optical correction. With aging, myopic patients, especially those with high myopia, suffer various ocular comorbidities such as cataract and maculopathy, which can lead to severe visual impairment (5). Although cataract is a reversible cause of blindness, it can be challenging to manage in myopic patients (6–8).

Along with the surging myopic population, the number of patients with cataract and myopia, particularly high myopia (severe myopia with an axial length [AL] ≥ 26 mm), being seen in the clinic is increasing. Previously, when cataract surgery was only performed to restore vision, the difficulty of managing patients with high myopia was mainly related to intraoperative risk factors (e.g., posterior capsular rupture) and postoperative complications (e.g., retinal detachment). In recent decades, the advent of premium intraocular lenses (IOLs) and switching from traditional cataract surgery to refractive cataract surgery have escalated the challenges encountered by cataract surgeons. Patients are becoming more demanding about their desired postoperative vision. Furthermore, the increased demand for surgical correction of refractive errors, including corneal-and lens-based refractive surgery, has greatly shifted the clinical features of cataract patients with myopia, presenting unprecedented challenges for ophthalmologists.

The purpose of this mini-review is to describe the impact of myopia on cataract patients against the background of the myopia epidemic, and to discuss new challenges that surgeons may encounter when planning refractive cataract surgery for myopic patients with cataract.

## Increasing prevalence of myopia

East and Southeast Asian countries, such as China and Japan, are myopia hotspots. The overall myopia rate in China was approximately 60% among schoolchildren, and exceeded 80% among high-school students (9–11). In Tokyo, 94.9% of teenagers were myopic according to a cross-sectional study conducted in 2017 (12). Although the current prevalence of myopia is lower in Europe and USA, the prevalence has doubled over the last decade (13–15). A landmark study by Professor Brien Holden predicted that, by 2050, almost half of the global population would be myopic and one in ten individuals would be highly myopic (1).

The COVID-19 pandemic affected countless aspects of people’s lives. Many governments implemented strict home quarantine policies to limit the spread of COVID-19. Although COVID-19 itself did not directly affect myopia, the associated lockdown, online schooling, and reduced outdoor activity advanced the onset and accelerated the progression of myopia (16). According to Ma et al., the mean myopia progression of children aged 8–10 years during the COVID-19 pandemic was significantly higher than that before the pandemic in 2018 (−0.93 vs. −0.33 diopters [D], *p* < 0.001) in China (17).

## Relationship between myopia and cataract

Myopia, in addition to the trouble seeing distant objects, may increase the risk of developing cataract. Population-based studies of different ethnicities have shown that myopia is associated with incident formation of nuclear cataract and posterior subcapsular cataract, with odds ratios ranging from 1.57 to 4.99 and from 1.34 to 1.93, respectively (18–23). Progression of mild myopia to high myopia is also associated with increased risk of cataract (20). Zhu et al. reported a significant correlation between high myopia and dark nuclear cataract, and suggested that the mechanism involved epigenetic downregulation of antioxidant genes in the lens in response to the increased oxygen tension caused by early vitreous liquefaction in highly myopic eyes (7, 8).

## Changing characteristics of the cataract population in the myopia epidemic era

Modern lifestyles have led to ever-increasing worldwide prevalence of myopia and high myopia, leading to changes in the characteristics of cataract patients. In China, nearly one-third of cataract patients presenting at tertiary hospitals have high myopia (8). Patients with high myopia often develop cataract much earlier than those without refractive errors, affecting many people in their forties.

Cataract surgery in working-age adults differs from that in older adults. Working-age adults have greater expectations of postoperative visual acuity, visual quality, quality of life, and even spectacle independence. Cataract surgery becomes more challenging when complicated with high myopia because surgery carries higher risk in highly myopic eyes (24). Young patients, regardless of whether implantation of a monofocal IOL or premium IOL is planned, should be informed of their higher risk of developing maculopathy or open-angle glaucoma to manage their expectations of cataract surgery outcomes.

Another important change in patient characteristics is that an increasing number of patients have a history of corneal refractive surgery (CRS). Since the pioneering experimental work on the surgical procedure for changing the corneal curvature by Father Waclaw Szuniewicz in 1948, CRS has evolved hugely, from radial keratotomy (RK) in 1970 to the introduction of the VisuMax femtosecond laser in 2007, which led to small incision lenticule extraction (SMILE). SMILE is a minimally invasive and accurate method for refractive correction, and its popularity has increased; over 7 million procedures have now been performed globally (25). Over 40 million eyes have undergone CRS (26), and many are likely to develop cataract in the following decades.

Implantable collamer lenses (ICLs) may be an alternative to CRS for myopic patients with an extremely high degree of myopia or thin cornea. The earlier ICL models were associated with development of lens opacities. A prospective clinical trial revealed that 47.4% of eyes developed anterior subcapsular opacities and 26.3% of eyes developed clinically significant cataract within a mean follow-up of 3 years after implantation of an ICL V2/V3/V4 in highly myopic patients aged >45 years (27). The latest model, V4c, has a central port allowing sufficient aqueous flow between the posterior and the anterior chamber to maintain the normal physiology of the anterior segment, and had a lower rate of lens opacity at a 5 years follow up (28). Longer-term follow-up is warranted.

## Emerging challenges and a paradigm shift in the management of cataract

The myopia epidemic has led to an unprecedented rise in the number of myopic and highly myopic patients. With aging, this population will be at very high risk of developing cataract. The popularity of subtractive and additive refractive surgery has caused many changes in these patients, and has led to greater demands on cataract surgeons, especially when planning refractive cataract surgery. Thus, new challenges are arising.

### Management of refractive surprises

To accomplish a perfect refractive cataract surgery, hitting the refractive target is of paramount importance. The first step is to acquire accurate ocular biometrics. Ocular parameters, including the anterior chamber depth, the AL, and the corneal curvature, often change after refractive surgery (29, 30). Thereinto, precise measurement of the corneal refractive power is the greatest challenge when managing myopic post-CRS eyes. Pitfalls lie in the variations of the anterior curvature in the central area and the altered relationship between the front and back surfaces of the cornea after CRS. The former makes it difficult to perform standard keratometry or corneal topography to measure the anterior corneal curvature accurately. The latter means the well-acknowledged standardized refraction index of a virgin cornea (1.3375) is unsuitable for predicting the total corneal power from the anterior curvature. Wang et al. reported that the ratio was 0.82 in normal eyes and 0.76 after photorefractive keratectomy/laser-assisted *in situ* keratomileusis (LASIK) (31). These pitfalls often lead to overestimation of the cornea refractive power and underestimation of the IOL power. To overcome these errors, new devices, such as Scheimpflug cameras, and optical coherence tomography (OCT) allow accurate measurement of the anterior and posterior curvatures.

Kyuyeon et al. reported high predictability of IOL power calculation following myopic laser refractive surgery using a Scheimpflug true net power of 4 mm (Haigis) or a Scheimpflug total corneal refractive power of 4 mm (Haigis); the latter had a mean arithmetic predicted error of 0.00 ± 1.09 D (32). The average values of the central 4 mm zone are more reliable than the readings obtained by standard keratometry (33). Furthermore, a recent study compared the accuracy of the Barrett True-K, Holladay 1 (D-K), and Haigis formulas for calculating IOL power, and the Barrett True-K formula was the most accurate in Chinese cataract patients with prior RK. However, even using the Barrett True-K formula, only 46.8% of eyes had an absolute error within 0.5 D (34). Thus, newer and more accurate IOL formulas are desired.

Errors in AL measurement previously contributed to unwanted refractive surprises in highly myopic eyes due to eccentric measurement to the depth of a posterior staphyloma, rather than to the fovea. Partial coherence interferometry or swept-source OCT have greatly improved the AL measurement error (35). However, cataract itself may also subtly contribute to AL measurement error, and a slight but significant reduction in the AL measurement after cataract surgery was reported (36). One hypothesis explaining the preoperative measurement error is that the real refractive index of the implanted lens is known, whereas the refractive index of the human lens could change according to the cataract grade (37). Considering that cataract is more frequent in myopic patients, lens opacity might assume another decisive role in AL accuracy. However, the refractive index of the human lens could be unreliable, and De Bernardo et al. proposed a linear regression formula to correct the preoperative AL to eliminate systematic error derived from this biometric (36). Additionally, the ICL itself may introduce a source of measurement error. Zhang et al. compared the biometrics before and after ICL V4c implantation and reported a slight but significant increase in AL measured using a Pentacam-AXL (OCULUS) and IOLMaster 500 (ZEISS) (38). They concluded that this change in AL did not affect the IOL power calculation using the Barrett Universal II formula. However, given that current IOLs are mostly designed with a 0.5 D interval, future comparative studies using an IOLMaster 700 (ZEISS) and other formulas are necessary to improve the measurement accuracy, especially if IOLs with a smaller diopter interval or individualized IOLs are developed.

After acquiring accurate biometrics, another challenge is precise prediction of the effective lens position (ELP) in post-CRS eyes, which largely depends on the appropriate selection of the IOL formula. Considering the altered corneal refractive power and anterior chamber depth after CRS, the classic IOL formulas may lead to inaccurate prediction of the ELP. For example, a flattened cornea causes a falsely shallow ELP, resulting in an insufficient IOL power and a hyperopic shift after surgery. This error might be avoided by using formulas that do not use the corneal power to infer the ELP, such as the Haigis-L and Shammas formulas (39, 40). Rosa et al. proposed a further advanced lens measurement approach that combines the R factor and AL × K (AL × mean keratometry) methods for post-CRS IOL power calculation with unknown preoperative parameters, and 79.41% of eyes had a refractive error of <1 D (41–43).

The available formulas can be categorized into two groups depending on whether they use historical data before CRS. Cataract surgeons now mostly prefer formulas that do not rely on historical data because most patients cannot provide this information, and even when these data are provided, they are not useful because the biometrics may change after CRS (36). Recently, Cione et al. proposed a multi-formula approach that is independent of historical data, and could be applied to different ranges of the corneal curvature and AL to improve the IOL power calculation after CRS (44). Their approach could improve the refractive outcomes of post-CRS eyes. Newer formulas based on big data and artificial intelligence (AI), such as the Hill-RBF and Kane formulas, have improved the accuracy of the IOL power calculation (45). However, it is unclear how these formulas work in post-CRS patients because no studies have been published. Thus, AI-based formulas designed for patients with history of CRS are required.

### Appropriate IOL selection

Implantation of a multifocal/extended-depth-of-focus (EDOF) IOL is a core aspect of refractive cataract surgery. Many studies have reported satisfactory visual outcomes for using different premium IOLs in myopic and highly myopic eyes and in eyes with a history of CRS. However, considering the increasing number of myopic patients and the popularity of CRS, every detail of cataract surgery needs to be ameliorated as much as possible.

The visual quality in terms of contrast sensitivity was reported to decrease in cataract patients after multifocal IOL implantation, possibly due to the distribution of light to different focal points (46). However, Bucci et al. reported that EDOF IOLs did not significantly decrease contrast sensitivity in cataract patients after LASIK compared with monofocal IOLs (47).

Despite concerns about visual quality, for highly myopic eyes with special anatomical features, ophthalmologists should select the appropriate IOL carefully, offering long-term stability. For example, Zhu et al. reported that IOL decentration was positively correlated with AL (48), which was probably due to incompatibility between the IOL size and the capsular bag size (6). For these eyes, a plate-haptic IOL might be more suitable than a C-loop haptic IOL because a plate-haptic IOL provides more friction as the main source of support. Due to the apparent inevitability of compromised IOL stability in highly myopic eyes, more knowledge of the tolerance to instability of different premium IOLs is necessary (49). Using the quick contrast-sensitivity function method, Guo et al. compared a trifocal IOL and an EDOF IOL, and suggested that the EDOF IOL showed better tolerance to IOL tilt (50).

Prior CRS might also cause irregular astigmatism and eccentric ablation is possible, which may contraindicate the implantation of multifocal/EDOF IOLs. However, myopic patients with a history of CRS usually express the greatest demand for achieving or continuing spectacle independence after cataract surgery. In this situation, careful planning of monovision cataract surgery and the use of a monofocal IOL might help (51), and crossed pseudophakic monovision could be planned in patients with refractive surprise after initial eye surgery to achieve spectacle independence if contraindications are to be avoided. Light-adjustable lens technology, offering postoperative refractive adjustments of IOL power, are theoretically appealing to patients with a history of CRS, whose preoperative IOL power calculation is relatively imprecise. However, as reported by Moshirfar et al., the precision of this technology is still compromised in patients with a history of CRS, emphasizing the need for further advancements in the technology of light adjustable lenses (52).

### Requirements for the surgical approach

Decreased corneal integrity caused by structurally weakening RK incisions poses a challenge to cataract surgeons. Zhang et al. analyzed the outcomes of phacoemulsification using different sizes of clear corneal incision in post-RK eyes and found that dehiscence of the RK incisions could be closed successfully by injecting an air bubble into the anterior chamber at the end of surgery (53). Alternatively, Wang et al. reported a smaller clear corneal incision or scleral tunnel incision might be safer (54).

It has been over 20 years since the first ICL implantation and gradually more patients with high myopia and an ICL are presenting at clinics for cataract surgery. Although several studies have shown that replacing the ICL and cataract with an IOL is safe (55, 56), the potential risk of corneal endothelium abrasion and the prolonged surgery time add to the difficulty of cataract surgery (57).

### Increasing frequency of complications after cataract surgery

As the number of myopic patients with cataract increase, surgeons are becoming more likely to encounter complications such as posterior capsular opacification (PCO) and retinal detachment.

PCO is the most common complication after cataract surgery and can be treated by Nd:YAG capsulotomy. In a study of 15,375 eyes, Lindholm et al. evaluated the 5 years cumulative probability of Nd:YAG capsulotomy after cataract surgery and found that low-diopter IOLs (5–16.5 D) implanted in myopic patients with cataract were associated with significantly greater risk (58). Another retrospective study of 500,872 operations confirmed that the risk of developing PCO was higher in eyes with an AL of >26 mm (59).

Retinal detachment is a severe complication of cataract surgery and can lead to irreversible visual impairment. Thylefors et al. and Lin et al. reported a strong correlation between pseudophakic rhegmatogenous retinal detachment and myopia (60, 61). The cumulative risk of retinal detachment development in highly myopic eyes after small incision coaxial phacoemulsification was 0.47, 0.71, 1.71, 2.59, and 3.28% at 3, 6, 15, 48, and 63–105 months, respectively (62). Thus, in myopic patients with cataract, especially those with high myopia, comprehensive preoperative fundus examination and regular follow-up are vitally important for protecting their vision.

### What is in store for the future?

SMILE has evolved into an established CRS for the correction of myopia. In the foreseeable future, the number of patients requiring cataract surgery after SMILE is expected to increase. Although recent literature suggests that SMILE technologies, unlike excimer-based procedures, achieved more favorable keratometric and aberrometric profiles, there are limited data for IOL power calculations after SMILE and a standard protocol has not been established. Only two comparative studies have examined the predictability of different IOL formulas after SMILE, and both suggested that ray tracing was superior to conventional formulas (63, 64). However, 20% of eyes had an absolute prediction error of >0.50 D, which may significantly compromise visual function. With accumulation of patient numbers and clinical data, it is worth investigating the accuracy of the newer generation of IOL formulas incorporating AI.

## Summary

The myopia epidemic is leading to an unprecedented increase in the global prevalence of myopia. Cataract surgery in myopic eyes, especially highly myopic eyes, is challenging, and the refractive surgery era has placed increasing demands on cataract surgeons, requiring highly accurate IOL power prediction to satisfy patients’ greater expectations. Knowledge of the characteristics of different IOLs may facilitate appropriate IOL selection for optimal long-term stability. Careful perioperative management of patients is also essential to reduce the risk of complications and to minimize related visual impairment.

## Author contributions

XZ and YL: conception or design of the work and critical revision of the manuscript. YD, JM, and WH: drafting the article. All authors contributed to the article and approved the submitted version.

## Funding

This work was supported by National Natural Science Foundation of China (82122017, 82271069, 82201161, 81870642, 81970780, 81670835), Science and Technology Innovation Action Plan of Shanghai Science and Technology Commission (19441900700, 21S31904900), Clinical Science and Technology Innovation Project of Shanghai Shenkang Hospital Development Center (SHDC12019X08, SHDC2020CR4078), Double-E Plan of Eye & ENT Hospital (SYA202006), Shanghai Municipal Key Clinical Specialty Program (shslczdzk01901).

## Conflict of interest

The authors declare that the research was conducted in the absence of any commercial or financial relationships that could be construed as a potential conflict of interest.

## Publisher’s note

All claims expressed in this article are solely those of the authors and do not necessarily represent those of their affiliated organizations, or those of the publisher, the editors and the reviewers. Any product that may be evaluated in this article, or claim that may be made by its manufacturer, is not guaranteed or endorsed by the publisher.
